# Lessons From the KK-Ay Mouse, a Spontaneous Animal Model for the Treatment of Human Type 2 Diabetic Nephropathy

**DOI:** 10.5812/numonthly.1954

**Published:** 2012-06-20

**Authors:** Yasuhiko Tomino

**Affiliations:** 1 Division of Nephrology, Department of Internal Medicine, Juntendo University Faculty of Medicine, Tokyo, Japan

**Keywords:** Models, Animal, Therapeutics, Diabetic Nephropathies

## Abstract

**Abstract:**

Diabetic nephropathy is a major cause of end-stage kidney disease (ESKD) in patients with type 1 and type 2 diabetes throughout the world. In human glomeruli, expansion of diffuse mesangial matrices, exudative lesions and/or segmental nodular sclerosis are pathological features of diabetic nephropathy. There have been many reports on the pathogenesis and treatment of type 2 diabetes using various animal models.

It appears that KK-Ay mice, especially in terms of their immunohistological findings, are a suitable animal model for human type 2 diabetic nephropathy. Many compounds have been reported to be advanced glycation end product (AGE) inhibitors such as aminoguanidine, angiotensin II receptor inhibitors and pyridoxamine, and these are useful in therapeutic interventions for reducing AGEs. Pyridoxamine ameliorates lipid peroxidation and insulin resistance in KK-Ay mice. Combination therapy with angiotensin converting inhibitors (ACE-I) and angiotensin II type 1 receptor blockers (ARB), including an ARB and 1,25-dihydroxyvitamin D3, i.e. anti-hypertensive and anti-reactive oxygen species effects, or with eicosapentaenoic acid (EPA), i.e. anti-microinflammation effect, have shown efficacy in the treatment of diabetic nephropathy in KK-Ay mice. It appears that KK-Ay mice are a useful spontaneous animal model for the evaluation of pathogenesis and treatment in patients with type 2 diabetic nephropathy.

## 1. Background

Diabetic nephropathy is a major cause of end-stage kidney disease (ESKD) in patients with type 1 and type 2 diabetes throughout the world. Almost 30% of diabetic patients in Japan develop nephropathy despite strict blood glucose and blood pressure control. It has been postulated that the initiation of this disease might be due to genetic factors. In human glomeruli, expansion of diffuse mesangial matrices, exudative lesions and/or segmental nodular sclerosis are pathological features of diabetic nephropathy. There are many progressive factors in patients with diabetic nephropathy, but no specific treatment for human diabetic nephropathy based on the initiative and progressive factors that have been found. Thus, it is important to determine both the pathogenesis and potential treatment options using various animal models of type 2 diabetes (Box).

**Box:** Spontaneous Mouse Model for Type 2 Diabetic Nephropathy

KK/Ta mouse
KK-Ay mouse
Diabetes (db/db) mouse
Obese (ob/ob) mouse
Fat (fat) mouse
Nagoya shibata yasuda (NSY) mouse
Tsumura suzuki obese diabetes (TSOD) mouse
Akita (Ins2) mouse
Tubby (tub) mouse
Agouti yellow (Ay) mouse
New Zealand obese (NZO) mouse

## 2. Objectives

The objectives of this review are 1) to show the characteristics of the KK-Ay mouse and 2) to introduce strategies for the treatment of type 2 diabetic nephropathy using this spontaneous animal model.

## 3. Characteristics of KK-Ay Mouse

### 3.1.Immunohistopathological Findings

The KK/Ta mouse, a model of type 2 diabetic nephropathy, originated from Japanese native mice as an inbred mouse by Kondo et al. in 1957 (1). This mouse strain is generally considered to be a polygenic disease model. Male KK/Ta mice spontaneously exhibit type 2 diabetes associated with hyperglycemia, glucose intolerance, hyperinsulinemia, mild obesity and microalbuminuria, conditions which are more severe than those found in the female (2-4). Since phenotypic characteristics of the KK/Ta mouse are not especially marked, the KK-Ay mouse was established by Nishimura et al. (5). This mouse was produced by transferring the yellow obese gene (Ay allele) into the KK/Ta mouse. KK-Ay mice exhibit obesity and hyperglycemia, including high levels of HbA1c and albuminuria (5). The KK-Ay mouse strain was established in 1969, and these mice are widely used as an experimental model for type 2 diabetes mellitus.

**Figure 1:**
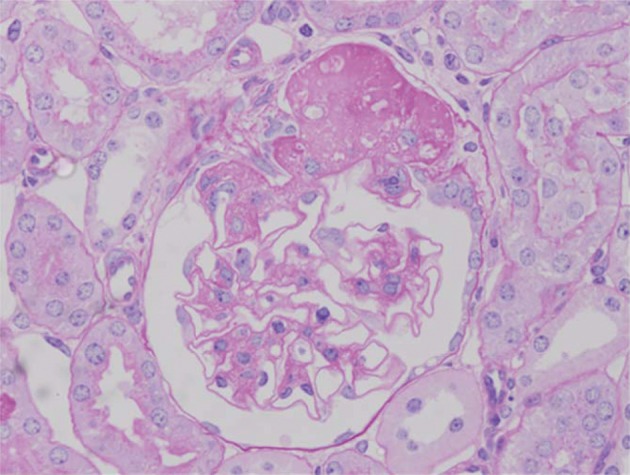
Light Microscopical Findings of a Glomerulus from a KK-Ay/Ta Mouse (PAS Staining, x400) The renal histological changes, i.e. diffuse mesangial expansion with mesangial cell proliferation and segmental sclerosis, in the KK-Ay/Ta mouse are more severe than those seen in the KK/Ta mouse.

**Figure 2:**
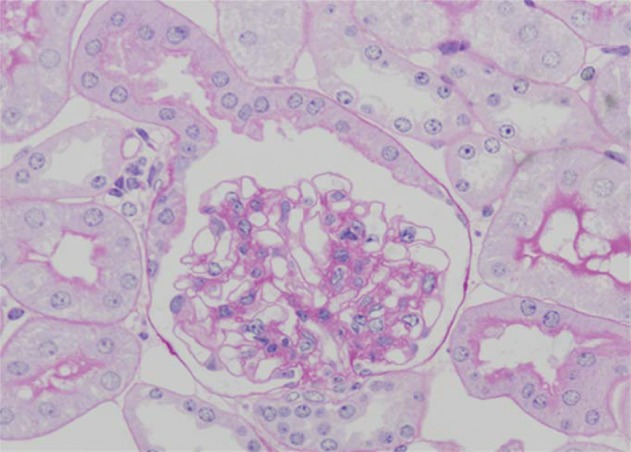
Light Microscopical Findings of a Glomerulus from a KK/Ta Mouse (PAS Staining, x400)

Pathological changes in the glomeruli of KK-Ay mice were consistent with those found in the early stages of human diabetic nephropathy (6). The renal histological changes, i.e. diffuse mesangial expansion with mesangial cell proliferation and segmental sclerosis in KK-Ay mice, are more severe than those which develop in KK/Ta mice (Figures 1 and 2) (Table) (6). Linear staining of IgG and diffuse thickening were observed in the glomerular capillary walls. Advanced glycation end products (AGE) and transforming growth factor-beta (TGF-β) protein appeared to be localized in the glomerular mesangial areas (7). The mice also develop renal lesions that closely resemble those seen in human diabetic nephropathy. It appears that KK-Ay mice, especially in terms of their histopathological findings, are a suitable animal model for the early stages of type 2 diabetic nephropathy.

**Table:** Glomerular Changes by Light and Electron Microscopy in KK/Ta and KK-Ay Mice

	**Age, Wk**	**Diffuse Mesangial Expansion**	**Segmental Sclerosis**	**GBM^a^ Thickening**
KK/Ta mouse	16-20	+^b^	-	+
KK-Ay mouse	20	++	-	++

^a^Abbreviation: GBM, glomerular basement membrane

^b^(-), negative; (+) , mild; (++), moderate

### 3.2.Podocyte Loss and Glomerulosclerosis

Morphometric analysis has contributed greatly to our understanding of diabetic nephropathy. Generally, the progression of nephropathy is associated with a reduction in the number of podocytes per glomerulus. Remaining podocytes are obliged to grow and to extend their foot processes in order to maintain the area that they cover. The authors previously reported that podocyte injury might provide additional prognostic information in patients with IgA nephropathy, the most common chronic glomerulonephritis, examining a small amount of renal biopsy tissue (8). Lemley et al. (9) reported podocytopenia (podocyte loss) and disease activity in patients with IgA nephropathy. However, the glomerular structure in type 2 diabetes has been studied less extensively even though this form of diabetes is a more common cause of ESKD. Macedo et al. (10) reported that control of hyperglycemia prevented the glomerular basement membrane (GBM) from thickening as well as reducing the number of podocytes in the early and late (12 months) stages of alloxan induced diabetic nephropathy. To determine the number of podocytes, the authors analyzed glomerular lesions in KK-Ay mice at 20 weeks of age through morphometric analysis. Levels of urinary protein were also measured. Glomerular enlargement and mesangial expansion were observed in KK-Ay mice at 20 weeks of age. The mean number of podocytes per glomerulus (NG pod) in diabetic KK-Ay mice was significantly lower than that seen in non-diabetic BALB/cA mice. Mean NG pod/ glomerular volume (GV) per glomerulus was also significantly decreased in the diabetic KK-Ay mice. Apoptotic bodies in the podocytes were observed by the tunnel method. The level of urinary albumin/creatinine (ACR) in the diabetic KK-Ay mice was significantly higher than that found in non-diabetic BALB/cA mice at 20 weeks of age. It appears that podocyte loss might induce albuminuria in KK-Ay mice (Ishikawa, et al. JDM, inpress).

### 3.3.Analysis of Candidate Genes

Pathogenesis and the development of human diabetic nephropathy involve genetic factors. Since human diabetic nephropathy is a heterogeneous disorder, the identification of the responsible gene loci is difficult. Aoki et al. (11) studied candidate gene loci for diabetic nephropathy using quantitative trait locus (QTL) analysis of the spontaneous animal model for diabetic nephropathy KK-Ay × normal BALB/cA F2 intercross mice. Urinary ACR, HbA1c and fasting body weights (FBW) at 8, 12, 16 and 20 weeks were examined in 270 (KK-Ay × BALB/cA) F2 intercross mice. Genotypes were investigated by QTL analysis using 86 microsatellite markers. ACR in mice at 20 weeks and ACR gain showed a suggestive linkage to chromosome 9 (LOD: 3.8 and 3.4 respectively; designed ACR-1). Gene loci contributing to HbA1c indicated a significant linkage to chromosome 7 (LOD: 5.8, 8.9) in mice at 8 and 20 weeks (designed HbA1c-1), and FBW indicated a significant linkage to chromosome 1 (LOD: 5.5, 5.2) in mice at 8 and 12 weeks (designed Fbw-1). At 20 weeks, the glomerular/Bowman’s capsule volume (G/B) ratio of F2 mice homozygous BB for D9Mit66 was significantly higher than that seen in the homozygous KK and heterozygous KB F2 progeny. Pancreatic islets in homozygous KK and heterozygous KB found in the D7Mit100 F2 progeny were larger than those in the homozygous BB F2 progeny. QTL analysis of the KK-Ay mice revealed several new loci contributing to diabetic nephropathy and related phenotypes. Thus, it appears that type 2 diabetes and diabetic nephropathy in KK-Ay mice have different genetic factors (11).

## 4. Approaches to the Treatment of Type 2 Diabetic Nephropathy

### 4.1.Anti-Glycation and Anti-Reactive Oxygen Species Effects of Pyridoxamine (K-163)

Non-enzymatic glycation has been implicated in the pathogenesis of diabetic nephropathy. There are multiple pathways for the formation of AGEs, including Nε-carboxymethyllysine (CML), Nε-carboxyethyllysine (CEL) and pentosidine, from glucose, antioxidation products of glucose, Schiff bases and Amadori products. The presence of AGEs is closely related to hyperglycemia and their pathobiochemistry could explain diabetic nephropathy (12). Specific AGEs, such as CML, are major products of glycoxidation reactions. In therapeutic interventions to reduce AGEs, many compounds have been reported to be effective AGE inhibitors, such as aminoguanidine, phenacyl thiazolium bromide, 2-isopropylidenehydrazono-4-oxo-thiazolidine-5-yl-acetanilide (OPB-9195), 2,3-diaminophenazine, vitamin C, vitamin E, angiotensin II receptor inhibitors and pyridoxamine (12).

Pyridoxamine was introduced by Khalifah et al. (13) as an inhibitor of AGE formation from Amadori products. Degenhardt et al. (14) reported that pyridoxamine inhibited AGE formation and retarded the development of diabetic nephropathy in streptozotocin treated rats, an animal model for type 1 diabetes mellitus. In 2007, my colleagues Tanimoto et al. (15), reported that the development of type 2 diabetic nephropathy could be prevented in KK-Ay mice by the use of pyridoxamine (K-163), an AGE inhibitor. AGEs have been associated with increased oxidative and nitrosative stresses in both in vitro and in vivo studies. Pyridoxamine, especially at 400 mg/kg body weight per day, improved levels of urinary ACR, fasting serum triglyceride (TG) and 3-deoxyglucosone (3DG) in KK-Ay mice. CML and nitrotyrosine accumulation in the glomeruli were decreased. TGF-β1 and laminin-β 1 messenger RNA expressions in the kidneys were significantly lower than those in the controls. The effect of pyridoxamine was related to improvement of CML and nitrotyrosine accumulation in the kidneys by the anti-AGE and/or antioxidant effects (15).

Furthermore, Murakoshi et al. (16) reported the pleiotropic effect of pyridoxamine on diabetic complications via CD 36 expression in KK-Ay mice. CD36 is an 88-kDa membrane glycoprotein belonging to the class B scavenger receptor family, which possesses one long extracellular loop between the two transmembrane domains. Pyridoxamine decreased levels of serum TG, especially VLDL, and fasting serum insulin. Accumulation of malondialdehyde (17), an advanced lipoxidation end product, in the pyridoxamine treated group was significantly lower than that in the untreated group. CD 36 accumulation and mRNA expression in the kidneys and adipose tissues in the treatment group were significantly higher than those in the untreated group. It appears that pyridoxamine ameliorated lipid peroxidation and insulin resistance in KK-Ay mice. This pleiotropic effect of pyridoxamine was related to CD36 expression in the kidneys and adipose tissues (16).

Clinically, the phase 2 clinical results demonstrated that the AGE blocker pyridoxamine is generally safe and well tolerated in patients with kidney disease caused by diabetes mellitus (18).

### 4.2.Anti-Hypertensive and Anti-Reactive Oxygen Species Effects

#### 4.2.1.Combination Therapy With an ACE (Angiotensin Converting Enzyme) Inhibitor (ACE-I) and an ARB (Angiotensin II Type 1 Receptor Blocker)

Systemic blood pressure is an important factor associated with the progression of diabetic nephropathy. It is generally considered that ACE-I and/or ARB have a renoprotective effect independent of systemic hypertension. Adenosine monophosphate activated protein kinase (AMPK) has a protective effect on lipid peroxidation. Adiponectin and AMPK might have a role in the pathogenesis of diabetic nephropathy. Blocking the renin-angiotensin system (RAS) increases adiponectin levels and reduces oxidative stress. Yamazaki et al. (19) examined lipid peroxidation via adiponectin and AMPK activation in the kidneys of KK-Ay mice with RAS inhibitors, such as enalapril and/or losartan. KK-Ay mice were given enalapril (2.5mg/kg/day) and/or losartan (25mg/kg/day), or hydralazine (25mg/kg/day) in their drinking water for 8 weeks starting at 8 weeks of age. They were divided into five groups as follows: enalapril 2.5 mg/kg/day treatment group (n = 5), losartan 25 mg/kg/day treatment group (n = 5), enalapril 2.5 mg/kg/day + losartan 25 mg/kg/day combination treatment group (n = 5), hydralazine 25mg/kg/day treatment group (n = 5) and tap water group as in the untreated group (n = 5). The urinary ACR, serum adiponectin and systemic blood pressure were measured as test parameters. Expressions of adiponectin, phospho-AMPK (p-AMPK) and phospho-acetyl CoA carboxylase (p-ACC) in the kidneys were evaluated through Western blot analyses. Pathological changes of glomeruli were also evaluated by light microscopy. Accumulations of Nε-carboxymethyllysine (CML), malondialdehyde (17) and 4-hydroxy-2-nonenal (4-HNE) in the glomeruli were evaluated by immunohistochemical analyses. Enalapril and/or losartan improved levels of urinary ACR with activation of adiponectin, p-AMPK and p-ACC in the kidneys. CML, MDA and 4-HNE expressions in the glomeruli were suppressed significantly with enalapril and/or losartan, especially in the combination treatment groups. In the hydralazine treatment group, the levels of urinary ACR and accumulation of CML/MDA did not improve although the systolic blood pressure decreased. Therefore, it was suggested that the renoprotective effects of ACE and/or ARB are related not only to systemic blood pressure but also to multiple other factors, some of which might be associated with adiponectin, AMPK and ACC activity. It appears that enalapril and/or losartan, especially in combination, inhibits the accumulation of CML/MDA/4-HNE in diabetic renal tissues. These effects might be related to lipid peroxidation via tissue-specific activation of adiponectin and AMPK (19).

Clinically, Jenning et al. (20) reported the results of a meta-analysis, which suggested that ACEI + ARB reduced 24-hr proteinuria to a greater extent in combination than with ACEI alone.

### 4.3.Combination Therapy With ARB and 1,25-dihydroxyvitamin D3

Several studies have suggested that the RAS is one of the major mediators for the progression of diabetic nephropathy. ARB is widely used in patients with diabetic nephropathy. However, ARB causes a compensatory renin increase due to the disruption of feedback inhibition in renin production. Recent studies have demonstrated that renin upregulates TGF-b1 and matrix proteins through the renin/prorenin receptors, independent of angiotensin II, which is regulated by extracellular-signal regulated kinase 1 and 2 (ERK1/2), a mitogen-activated protein kinase (MAPK) (21, 22). Kaneshiro et al. (23) reported that the human renin/prorenin receptor elicits slow progressive nephropathy through angiotensin II independent MAPKs activation, and initiation of glomerulosclerosis with increased TGF-b1 expression in human renin/prorenin receptor overexpressed transgenic rats.

On the other hand, calcitriol, 1,25-dihydroxyvitamin D3 (1,25(OH)2D3) and its analogs have been shown to have therapeutic potential in attenuating experimentally induced kidney diseases (24-29). Schwarz et al. (25) reported that calcitriol treatment suppresses the progression of glomerulosclerosis and albuminuria in subtotally nephrectomized rats. Makibayashi et al. (26) reported that 22-oxacalcitriol, one of the 1,25(OH)2D3 analogs, has been shown to reduce both mesangial cell proliferation and glomerulosclerosis in anti-Thy-1 glomerulonephritis in rats. 1,25(OH)2D3 is a negative endocrine regulator of RAS and suppresses renin biosynthesis (27, 28). These studies provide a molecular basis to explore the potential of 1,25(OH)2D3 as a renin inhibitor to control RAS (27). Therefore, it is postulated that combination therapy with ARB and 1,25(OH)2D3 is more effective. In fact, Zhang et al. (30) reported that combination therapy with ARB and vitamin D analog markedly ameliorates diabetic nephropathy in streptozotosin treated diabetic mice. However, the mechanisms have not been fully determined in type 2 diabetic nephropathy.

Ohara et al. (31) examined the preventive effects of combination therapy with an ARB and 1,25(OH)2D3 in diabetic nephropathy of KK-Ay mice. KK-Ay mice were divided into four groups as follows: ARB group, 1,25(OH)2D3 group, combination group and control group. The urinary ACR was measured for phenotypic characterization. Renin, p-ERK1/2 and TGF-b1 protein expressions in the renal tissues were evaluated. The levels of urinary ACR in the combination group were significantly lower than those in the ARB group or control group. Renin protein expressions on renal tissues in the ARB group were significantly increased compared with those found in the control group. In the 1,25(OH)2D3 group or combination group, renin protein expressions were significantly decreased compared with those in the ARB group. The protein expressions of p-ERK1/2 in the combination group were significantly decreased compared with those in the control group or ARB group. The protein expressions of TGF-b1 in the ARB group and/or combination groups, especially the combination group, were significantly decreased compared with those of the control group. It appears that combination therapy with ARB and 1,25(OH)2D3 improves the levels of urinary ACR by suppressing the compensatory renin increase in type 2 diabetic nephropathy. These effects might be related to the suppression of renin-ERK1/2-TGF-b1, which may or may not depend on angiotensin II (31).

Clinically, Zeeuw et al. (17) reported that the addition of 2 μg/day paricalcitol to renin angiotensin aldosterone system (RAAS) inhibition safely lowers residual albuminuria in patients with diabetic nephropathy, and this could be a novel approach to lowering the residual renal risk in diabetes.

### 4.4.Anti-Microinflammatory Effect of Eicosapentaenoic Acid (EPA)

Previous studies reported that eicosapentaenoic acid (EPA) was effective against all renal diseases including diabetic nephropathy. EPA is one of the n-3 polyunsaturated fatty acids (PUFA) present in fish oil. It was found that EPA has many effects including anti-thrombotic, hypolipidemic, anti-atherogenic, anti-inflammatory and anti-mitogenic actions. Monocyte chemoattractant protein-1 (MCP-1) is a regulating macrophage recruitment protein, which is up-regulated in patients with diabetic nephropathy. In KK-Ay mice injected with EPA ethyl ester (1g/kg/day), Zhang et al. (32) and Hagiwara et al. (30) reported that EPA improved type 2 diabetic nephropathy in KK-Ay mice by decreasing hypertriglyceridemia, glucose tolerance and albuminuria. Glomerular mesangial matrix expansion and segmental sclerosis, as well as interstitial fibrosis were markedly decreased by EPA treatment. Diabetes induced up-regulation of MCP-1 and TGF-beta expressions were inhibited by EPA, together with a reduction of glomerular macrophage infiltration and oxidative stress. It appears that EPA might be an effective therapeutic agent for diabetic nephropathy (33).

Clinically, Lee et al. (34) examined the association between dietary n-3 long-chain polyunsaturated fatty acids (n-3LC-PUFAs), incident albuminuria and changes in urinary albumin excretion rates (UAER) over time in type 1 diabetes. Dietary n-3LC-PUFAs appear to be inversely associated with the degree but not with the incidence of albuminuria in type 1 diabetes.

In conclusion, it appears that KK-Ay mice are a useful spontaneous animal model for the evaluation of pathogenesis and treatment in patients with type 2 diabetic nephropathy. Confirmation that the treatments studied in the KK-Ay mouse are effective in patients with type 2 diabetic nephropathy will be necessary in the future.
